# Local-global functional gradients of the thalamus capturing different aspects of thalamic structure and function

**DOI:** 10.1162/IMAG.a.1094

**Published:** 2026-01-12

**Authors:** Hua Xie, Venkata Sita Priyanka Illapani, William D. Gaillard, Leigh N. Sepeta, Seok-Jun Hong

**Affiliations:** Center for Neuroscience Research, Children’s National Research Institute, Children’s National Hospital, Washington, DC, United States; Department of Neurology & Rehabilitation Medicine, The George Washington University School of Medicine and Health Sciences, Washington, DC, United States; Departments of Psychiatry & Behavioral Health, The George Washington University School of Medicine and Health Sciences, Washington, DC, United States; Department of Biomedical Engineering, Sungkyunkwan University, Suwon, South Korea; Center for Neuroscience Imaging Research, Institute for Basic Science, Suwon, South Korea

**Keywords:** thalamus, thalamic function, thalamic anatomy, functional MRI, local-global functional connectome, gradient

## Abstract

The thalamus, a core hub positioned deep in the center of the brain, has a highly complex inter-regional communication pattern. The thalamic functional connectome has received increasing attention, showcasing its pivotal role in various high-order cognitive processes and development. However, thalamic connectome profiles across different spatial scales have not been systematically investigated. More specifically, it is unknown how each thalamic voxel is functionally connected to one another on a local level, and how thalamic voxels are functionally embedded within large-scale cortical systems on a global level. Leveraging the recent connectopic gradient mapping techniques and incorporating our methodological updates, we shed light on the thalamic local-global connectome profiles by characterizing their respective functional gradients. We show that the local gradients of the thalamus primarily reflect its internal anatomical configuration, while its global counterparts predominantly reflect the thalamus’s inherent role in supporting various cognitive functions.

## 1 Introduction

The thalamus is a core subcortical structure that is connected with distributed cortical regions, acting as a crucial hub and gateway for cortical networks. While the thalamus was previously thought as of a relay that transfers sensory information to the cortex, recent studies increasingly highlight its role in orchestrating the integration of neural information between cortical networks (Hwang et al., 2017; Shine et al., 2023), critical for various high-order cognitive processes, such as executive function, memory, and attention (Aggleton & O’Mara, 2022; Hwang et al., 2022; Steiner et al., 2020).

Because of its complex inter-regional communication patterns, recent research has focused on the network embedding of the thalamus within the whole brain to elucidate its large-scale organizational principles. Although the anatomical distribution of thalamocortical connections is well-characterized (Behrens et al., 2003), its functional counterpart remains incompletely understood, primarily due to the lack of scalable analytical methods, especially for the whole brain. One prominent approach has been to analyze the functional connectome, often measured using functional connectivity (FC), which captures the temporal correlation between the activity of different brain regions as a proxy for functional coupling. Yet, the high dimensional nature of FC, primarily due to fine-grained brain parcellation schemes, makes it challenging to summarize the network embedding patterns into more parsimonious principles.

The connectopic gradient mapping technique (Haak et al., 2018) has been recently introduced to address this issue, providing an interpretable metric to describe modes of brains’ functional organization. Instead of delineating discrete functional boundaries, this technique characterizes the similarity of FC profiles within a given region to describe smoothly shifting connectome modes, known as functional gradients. For the last decade, this novel analytical perspective has refueled discussion about principles underlying functional brain organization (Bernhardt et al., 2022; Petersen et al., 2024) and spurred biomarker development (Hong et al., 2019; Hong et al., 2020).

This technique has accelerated the study of the thalamus. For example, Park and colleagues used the connectopic mapping to examine thalamocortical connectivity in typically developing children, revealing anterior-to-posterior and superior-to-inferior gradients. Their work demonstrated the pivotal role of the thalamus in the hierarchical differentiation of large-scale functional networks as a functional scaffold (Park et al., 2024). Yang and colleagues examined the thalamic gradients in adult brains and identified lateral-to-medial and anterior-to-posterior gradients, which were found to be related to thalamic structure and cognition, respectively (Yang et al., 2020). Tian and colleagues noted three major thalamic gradients along the antero-posterior, mediodorsal, and ventrolateral axes within the subcortex, possibly related to organizational differences between subgroups of thalamic nuclei (Tian et al., 2020). In addition to studies of healthy individuals, thalamic gradients have been also examined in clinical populations. For example, Feng and colleagues examined thalamic gradients in pediatric patients with temporal lobe epilepsy and found patients’ first two thalamic gradients showed increased connectivity to the bilateral basal ganglia and mesial temporal regions as compared to typically developing controls (Feng et al., 2025). Moreover, Fan and colleagues observed increased segregation of thalamic functional gradients in early-onset schizophrenia patients, which may reflect the potential altered thalamocortical interactions with unimodal and transmodal networks (Fan et al., 2023).

Despite recent progress, the relationship between the local and global organizational principles of the thalamic functional connectome remains unclear. More specifically, it is not fully understood how functional connectivity is organized locally within the thalamus and how this internal organization is topographically related to its global embedding within the global large-scale cortical systems. To fill this remaining gap, we have utilized existing connectopic mapping techniques and explored variants to systematically investigate the thalamic gradients using two large-scale public datasets. We aimed to demonstrate the neurobiological relevance of the thalamic functional gradients and shed light on the nature of the local-global connectome profiles of thalamus. Building on previous work that has associated thalamic gradients with cognition, geometry, and cytoarchitecture (Müller et al., 2020; Tian et al., 2020; Yang et al., 2020), we further examined how the local-global gradients differentially reflect those factors.

## 2 Methods

### 2.1 Participants

We examined two large-scale publicly available resting-state datasets, that is, Human Connectome Project young adults (HCP-YA) and its early lifespan version (HCP-Development, HCP-D) (Somerville et al., 2018; Van Essen et al., 2013). Both datasets are collected using a very similar protocol except for minimal changes optimized to the study populations. Details of the image acquisition protocols can be found in (Harms et al., 2018). The HCP-D dataset consists of 652 typically developing children and adolescents between the age of 5 to 21. We kept 598 participants based on the following inclusion criteria: low motion (mean framewise displacement <0.4 mm), with four runs, without focal anomalies, and successful segmentation and surface reconstruction. For the HCP-YA dataset, we randomly chose a subset of 598 participants between the age of 22 to 35 based on the above-mentioned inclusion criteria from the S900 release to match the sample size of the HCP-D dataset. The demographic information of the included participants can be found in Supplementary Table S1. All participants provided written informed consent and assent, and parents of those under 18 years provided written informed consent on behalf of their child.

### 2.2 Preprocessing

For the HCP-D dataset, a standard preprocessing pipeline (fMRIPrep v24.0.0 (Esteban et al., 2019)) was used to preprocess the resting-state data. Briefly, the functional data were registered to T1w structural data after susceptibility distortion correction, slice-timing correction, and motion correction. We applied a band-pass filter between 0.01 and 0.08 Hz and removed the linear and quadratic trends. We regressed out following nuisance regressors, that is, six motion parameters, mean signal of ventricular and white matter signal, and their temporal derivatives. The denoised data were concatenated across runs and then projected to the standard grayordinates space (MNINonLinear_fs_LR_32k space) in CIFTI format using Ciftify tools (https://github.com/edickie/ciftify (Dickie et al., 2019)). The 91,282 standard-mesh grayordinates space consisted of 2-dimensional cortical surface mesh (left and right) with average 2 mm vertex spacing, and 3-dimensional subcortical volumetric parcels (such as thalamus) based on FreeSurfer subcortical atlas with 2 mm voxel resolution in the MNI space (Glasser et al., 2013). The left and right thalamus has 1288 and 1248 voxels, respectively. Following the projection, the denoised data underwent spatial smoothing across each surface independently using a 6 mm full width at half maximum (FWHM) Gaussian kernel.

For the HCP-YA dataset, the ICA-FIX (Salimi-Khorshidi et al., 2014) preprocessed grayordinate timeseries with grayordinate-constrained smoothing (2 mm), which were z-scored within each run and subsequently concatenated across runs. Details of preprocessing pipeline of the HCP-YA dataset can be found in (Glasser et al., 2013). Given the lack of an optimal preprocessing pipeline for the gradient analysis, we chose two different preprocessing and commonly used preprocessing pipelines. This approach allowed us to ensure our findings were not specific to certain data analytic decisions (such as smoothing) as cautioned by Watson and Andrews (2023), and to focus on the consensus results for increased reproducibility and rigor (Alahmadi, 2021).

### 2.3 Overview of the original connectopic mapping technique

**Fig. 1.**
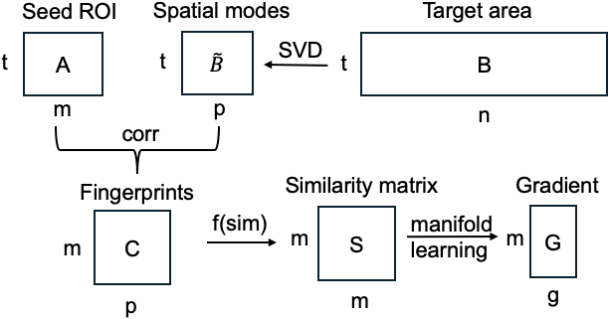
A schematic of the original connectopic mapping technique (Haak et al., 2018).

We first give an overview of the original connectopic mapping technique (Haak et al., 2018; Fig. 1). Please note that we provide the mathematical details of each step of the original implementation to subsequently explain our novel algorithmic modifications. Following preprocessing, the below steps were conducted to obtain functional gradient (i.e., primary modes of topographic organization for the seed-based connectivity).

1) Timeseries extraction. The voxelwise timeseries within the seed ROI are separately extracted from left and right thalamus defined based on the FreeSurfer subcortical atlas, termed as $A\in {\mathbb{R}}^{t\times m}$ , where *m* is the number of voxels within the seed ROI and *t* is the number of time points. We also extract the timeseries from a target area, termed as $B\in {\mathbb{R}}^{t\times n}$ , where *n* is the number of voxels of the target region. We combined the left and right cerebral hemisphere from FreeSurfer segmentation as the target area.

2) Singular value decomposition (SVD). For the purposes of computational efficiency, SVD is applied to the larger matrix B that contains timeseries from neocortex such that $B=U\Sigma V^{T}$. SVD results in p spatial uncorrelated components or spatial modes: $\tilde{B}=U\Sigma \in {\mathbb{R}}^{t\times p}$ , where p=t−1 .

3) Spatial modes computation. Then timeseries of the seed ROI are correlated against those of the spatial modes to obtain a matrix $C=corr(A,\tilde{B})\in {\mathbb{R}}^{m\times p}$ , where each column in the matrix C represents the connectivity ﬁngerprint, that is, functional connectivity (FC) profile between m seed voxels to p spatial modes.

4) Voxel-to-voxel similarity matrix calculation. A voxel-to-voxel similarity matrix $S\in {\mathbb{R}}^{m\times m}$ is calculated by comparing the connectivity ﬁngerprints of all seed voxels, and some common similarity measures include η^2^ coefficient (Cohen et al., 2008) and cosine similarity. Voxels with similar FC profiles produce high similarity.

5) Manifold learning. A manifold learning algorithm (e.g., Laplacian eigenmap) is applied to the similarity matrix to obtain ROI’s functional gradients $G\in {\mathbb{R}}^{m\times g}$ , where g is the number of gradients to be kept. The group-level gradients are calculated by averaging the individual-level similarity matrices S, and the individuals’ gradient is subsequently aligned to their corresponding group-level gradient using Procrustes alignment.

### 2.4 Modifications to the original connectopic mapping technique

**Fig. 2.**
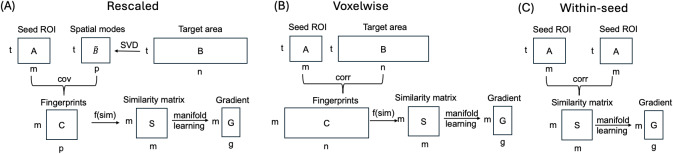
Schematics of the modified connectopic mapping technique: (A) rescaled, (B) voxelwise, and (C) within-seed FC implementation.

SVD reduces the dimensionality and retains only the most significant singular values and eigenvectors, improving computational efficiency. The subsequent correlation operation to calculate fingerprints normalizes the variance of each column of the dimensionality-reduced matrix $\tilde{B}$ , treating each spatial mode equally and ignoring the differences in associated singular values. Therefore, we proposed to rescale the correlation matrix accordingly so that $C=corr(A,\tilde{B})\times \Sigma$ . To better understand the difference between the original and rescaled implementations, we calculated two additional gradients, that is, a voxelwise gradient and a within-seed gradient. For the “voxelwise” gradient, we removed the SVD step and directly calculated the voxel-to-voxel FC between all seed and target voxels so that C=corr(A,B) . The voxelwise variant serves as the ground truth to benchmark the original and rescaled implementations that is supposed to truly capture the whole-brain embedding of the ROI. For the “within-seed FC” variant, we skipped the step that calculates fingerprints, and directly used within-seed FC to calculate gradients, which reflects *local* connectivity organization, for example, how the voxels are connected to each other within the thalamus. The schematics of the modified gradient implementations are shown in Figure 2.

While group-level gradients are calculated in the same way by averaging individuals’ similarity matrix, we modified the individual-to-group gradient alignment procedure, as the individual-level gradients may not be ordered in the same way as the group-level gradients. We matched individuals’ gradients to the group-level gradients using the Hungarian algorithm (https://www.mathworks.com/matlabcentral/fileexchange/20652-hungarian-algorithm-for-linear-assignment-problems-v2-3), which maximizes the overall similarity between group-level and individual-level gradients. We then proceeded to align individuals’ gradients to their matched group-level gradients using Procrustes alignment. In addition, for generalizability, we also examined all three manifold learning techniques offered by the BrainSpace toolbox (https://github.com/MICA-MNI/BrainSpace (Vos de Wael et al., 2020)). These three manifold techniques include Laplacian eigenmap (LE), diffusion map embedding (DE), and principal component analysis (PCA). Following previous gradient literatures (Dong et al., 2021; Hong et al., 2019; Huang et al., 2023; Margulies et al., 2016; Pini et al., 2025; Serhan et al., 2025), we used the default sparsity threshold of 0.9 (i.e., keeping only the top 10% of connections per row). We then focused on the first three gradients given their relatively stability, as the average spatial similarity between individual- and group-level gradients exceeded 0.5.

### 2.5 Relationship between gradients and thalamic geometry

Given recent observations that highlight that the critical role of the geometry of brain play in reshaping the brain’s inter-regional connectivity (Pang et al., 2023), we examined how thalamic gradients are spatially constrained by the local anatomy of the thalamus. We used representational similarity analysis (Kriegeskorte, 2008) to determine the influence of geometry of the thalamus on the resulting gradients. We first computed a voxel-to-voxel dissimilarity matrix of the first three gradients using Pearson distance for each participant, which was then correlated against the voxel-to-voxel physical distance matrix using Euclidean distance using their MNI coordinates. Moreover, we examined how thalamic gradients track the thalamic nuclei by entering the first three group-level gradients into a linear discriminant analysis model using MATLAB function fitcdiscr.m to predict the label of thalamic nuclei of AAL3 atlas (Rolls et al., 2020), which includes anterior, lateral, ventral, intralaminar, medial, and posterior nucleus. The discriminant analysis was conducted for the left and right thalamus separately, and no confound adjustment was performed. We repeated the analysis 20 times and reported the 5-fold cross-validated classification accuracy.

### 2.6 Relationship between gradients and thalamic cytoarchitecture

The thalamus exhibits topographical organization along a core–matrix cytoarchitectural gradient, as determined by relative distribution of core-like or matrix-like neurons (Harris et al., 2019; Jones, 2001). We assessed the spatial similarity between the thalamic gradients and the core–matrix cytoarchitectural gradient, using the relative expression of PVALB and CALB1 genes—differentially enriched in core and matrix neurons—as a proxy for the core–matrix gradient (Müller et al., 2020), available at (https://github.com/macshine/corematrix).

### 2.7 Gradient-based individual difference prediction

To examine the utility of the obtained gradients as a biomarker, we entered the first three gradients from manifold learning techniques into a LASSO model to predict various individual differences. We examined nine individual difference metrics and grouped them into 1) four local anatomical metrics derived from FreeSurfer (left and right thalamus volume and both of these volumes normalized by intracranial volume); and 2) five global cognitive metrics from HCP-D and HCP-YA datasets (Picture Sequence Episodic Memory Test, Pattern Comparison Processing Speed Test, Dimensional Change Card Sort Test, Flanker Inhibitory Control and Attention Test, Total Cognition, all of which are age adjusted). These tests measure a wide range of cognitive abilities, such as executive function, episodic memory, processing speed, cognitive flexibility, inhibition control and attention, for all of which the thalamus is critically involved (Aggleton & O’Mara, 2022; Hwang et al., 2022; Steiner et al., 2020). The first three individual-level gradients were z-scored within individuals and then entered into a LASSO model using MATLAB function fitrlinear.m to predict individual difference metrics. We repeated the 10-by-10-fold nested cross-validation 20 times for robustness. The inner loop determined the optimal sparsity hyperparameter which was used in the outer loop, and we reported cross-validated prediction performance of the outer loop in terms of the Spearman correlation between true vs. predicted metric.

## 3 Results

### 3.1 Different connectopic mapping implementations capture thalamic local and global connectome profiles

Besides the original connectopic mapping implementation, we implemented three additional variants, that is, the rescaled, voxelwise, and within-seed FC implementation. The voxelwise implementation uses voxel-to-voxel FC between the thalamus and the neocortex without SVD, which serves as a lossless representation for the global thalamocortical connectome profiles. The within-seed FC implementation captures the local connectome profiles within the thalamus.

**Fig. 3.**
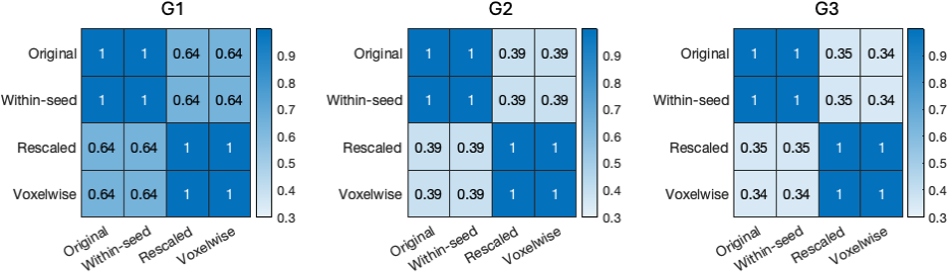
Mean similarity of unaligned individual-level gradients across the four connectopic mapping implementations for the first three gradients.

We then compared the individual-level gradients by computing the similarity of the unaligned individual-level gradients from four implementations, to ensure that the comparison is unaffected by the alignment to the group-level gradients. Figure 3 shows the similarity of four implementations, averaged across all HCP-D participants and three manifold learning techniques (see Supplementary Table S2 for more detailed breakdown). We can observe that the gradients obtained using the original implementation were identical to those using the within-seed FC implementation, suggesting the original connectopic mapping captures the local connectome profiles within the thalamus. The gradients based on the rescaled variant mirrored those from the voxelwise variant, suggesting the rescaled variant truly captures the thalamocortical connectome profiles and reflects the whole-brain embedding of the thalamus on the global scale. Therefore, we named the gradients from the original implementation the local gradients, and termed the gradients from the rescaled variant the global gradients.

In addition, we performed a simulation analysis to validate our empirical observations by randomly selecting seed and target areas to demonstrate our findings remained unchanged regardless of the choice of seed or target area. The simulation analysis and the mathematical proof are provided in the Supplementary Materials.

**Fig. 4.**
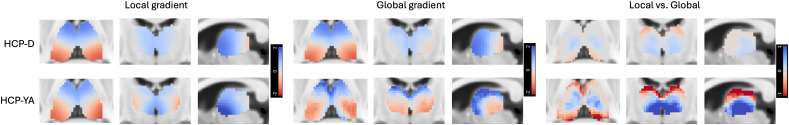
Visualization of the primary group-level gradients obtained with diffusion map embedding for the local, global and the difference between the two gradients (local-global). For visualization and comparison purposes, all gradients were z-scored.

As shown in Figure 4, we visualized the primary group-level local gradients using the original implementation and the global gradient using the rescaled variant. We can observe that on the group level, while the primary gradients were more comparable for the HCP-D dataset than the HCP-YA dataset, all primary gradients in general followed an anterior-to-posterior direction. Moreover, we also compared the local and global gradients by directly subtracting the two after converting gradient values to z-scores (Fig. 4, right panel). We found across two datasets, the difference patterns also were comparable, with a spatial correlation of 0.714. Greater discrepancy was observed in the second and third gradients between local and global gradients. The detailed similarity for the first three local-global gradients can be found in Table 1. See Supplemental Figure 2 for the variance explained of the first three gradients.

**Table 1.** Similarity of the first three group-level local and global gradients via three manifold learning techniques.

	HCP-D			HCP-YA		
	G1	G2	G3	G1	G2	G3
DE	0.993	0.992	0.782	0.763	0.710	0.140
LE	0.851	0.764	0.583	0.644	0.241	0.039
PCA	0.826	0.754	0.753	0.345	0.361	0.241
Avg	0.890	0.836	0.706	0.584	0.437	0.140

Diffusion map embedding (DE), Laplacian eigenmap (LE), and principal component analysis (PCA).

### 3.2 Anatomical constraints on thalamic gradients

**Fig. 5.**
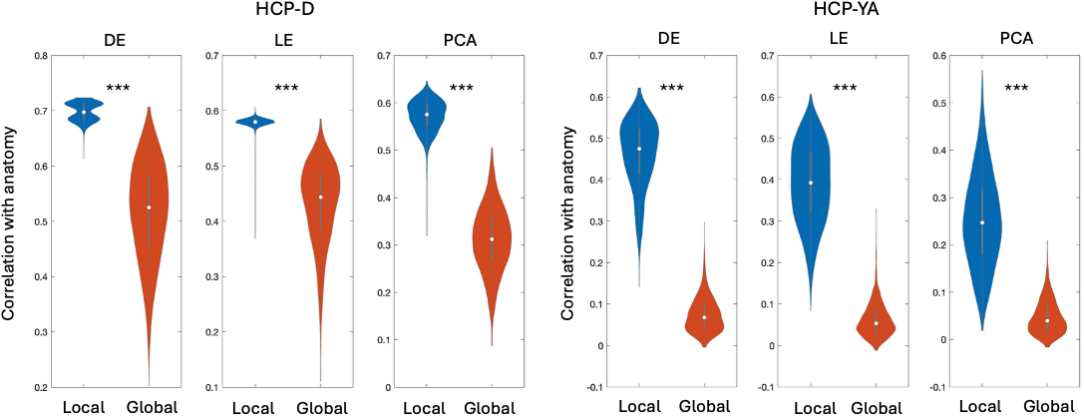
Violin plots of the anatomical constraints on the first three individual-level gradients derived using diffusion map embedding (DE), Laplacian eigenmap (LE), and principal component analysis (PCA). ***p < 0.001.

To understand how different gradients are constrained by the thalamic geometry, we performed a dissimilarity analysis of individuals’ first three gradients by correlating gradients’ voxelwise dissimilarity matrix with the voxelwise Euclidean distance matrix. As shown in Figure. 5, we found across two datasets and three manifold learning methods, the local gradients were significantly more constrained by the anatomy of the thalamus than the global gradients, which was evidenced by the significantly higher correlation between the spatial distance and gradient dissimilarity (*p* < 0.001, paired t-test). It is worth noting that while the spatial constraint on global gradients was much weaker, the correlation was still significantly higher than the chance-level correlation based on permutation tests.

**Fig. 6.**
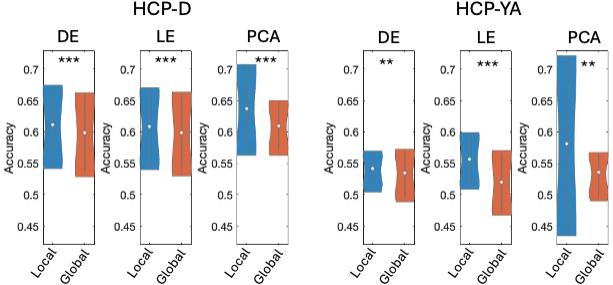
Violin plots of the thalamic nucleus prediction with the first three local-global thalamic gradients derived using Laplacian eigenmap (LE), diffusion map embedding (DE), and principal component analysis (PCA). ***p < 0.001; **p < 0.01.

Moreover, we performed a discriminant analysis using the first three gradients to predict labels thalamic nuclei as defined by the AAL3 atlas. Across 20 repetitions, we found that while both gradients far exceeded the chance-accuracy (30.99% based on permutation tests), local gradients significantly better predicted the label of the thalamic nuclei (all *ps* < 0.01, Wilcoxon signed-rank test, Fig. 6).

### 3.3 Local and global gradients differentially capture individual differences in local thalamic characteristics and cognition

**Fig. 7.**
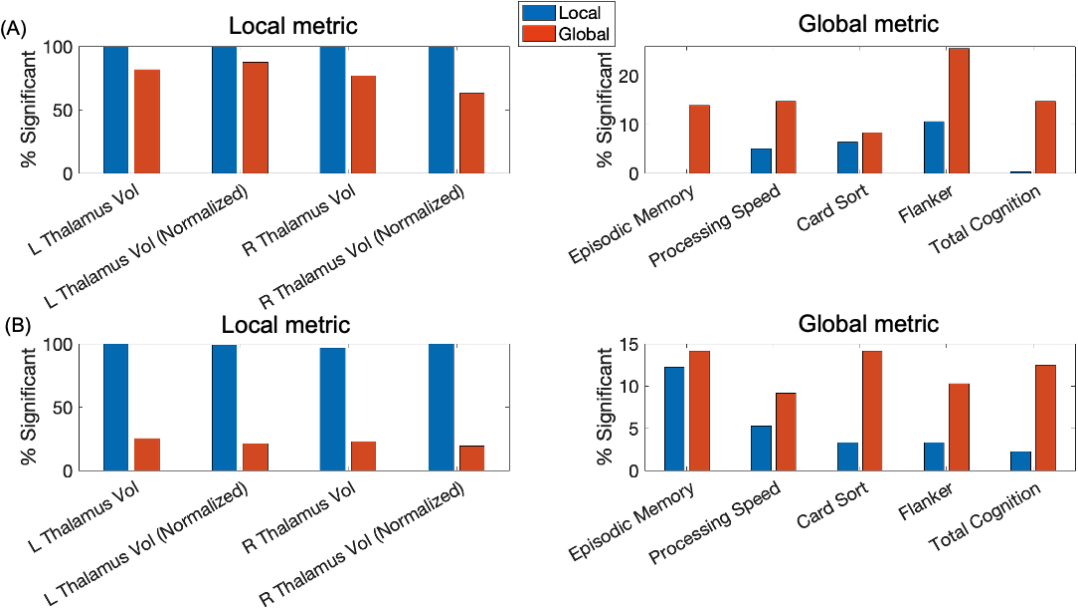
Comparing individual difference predictive performance predicting global cognitive and local anatomical metrics using the first three thalamic gradients from (A) HCP-D and (B) HCP-YA dataset. Y-axis: the percentage of significant positive correlation for each individual difference metric (*p_corrected_* < 0.05, Bonferroni corrected).

To examine what information local and global gradients encode, we fed the first three functional gradients, obtained with manifold learning technique of DE, LE, and PCA, to a LASSO model to predict individual difference in cognition and local anatomy, a process we repeated 20 times. As shown in Figure 7, we found the global gradients led to significantly more successful predictions (as defined by *p_corrected_* < 0.05, Bonferroni corrected) in terms of predicting global cognitive metrics (*p* < 0.001, Mann-Whitney U test), while local gradients significantly outperformed the global counterparts in terms of predicting local anatomical metrics (*p* < 0.001, Mann-Whitney U test).

**Fig. 8.**
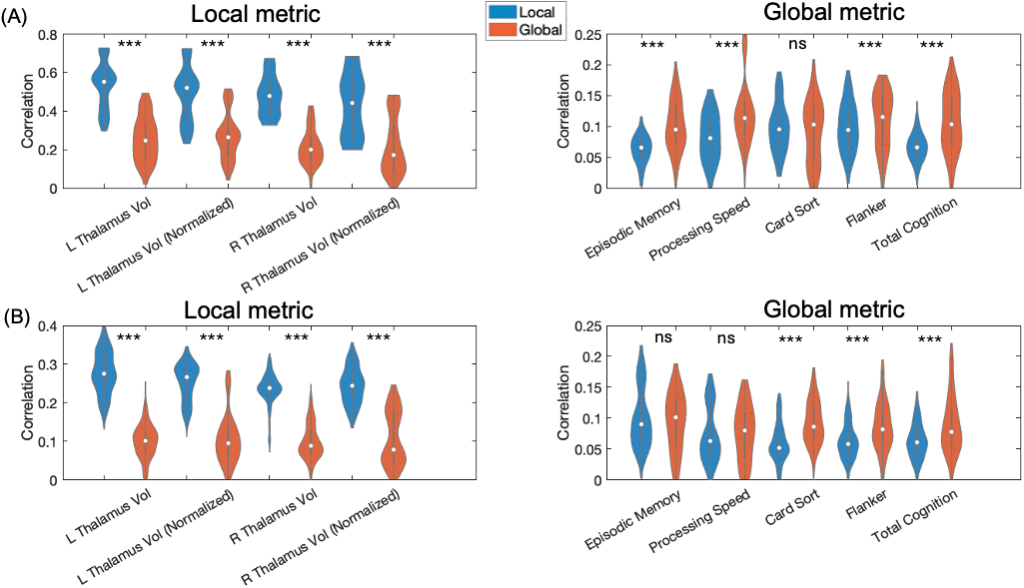
Local and global individual difference prediction using the first three local-global thalamic gradients from (A) HCP-D and (B) HCP-YA dataset. Y-axis: spearman correlation between true and predicted metric. ***p < 0.001; ns, not significant.

Looking more closely at each phenotype (Fig. 8), we noticed that out of five cognitive phenotypes, the global gradients significantly better predicted four and three cognitive phenotypes in the HCP-D dataset and the HCP-YA dataset, respectively. The local gradients also achieved significantly better performance in predicting all local anatomical metrics (all *ps* < 0.001, Wilcoxon signed-rank test). To check if any of the first three gradients drove the prediction, we used Kruskal-Wallis test on the first three gradients’ prediction accuracy, and found they similarly contributed to the local or global individual difference prediction.

We also compared the neural signatures predicting local anatomical and global cognitive metrics. As for the anatomy-predictive patterns, the local gradients yielded much more consistent predictive patterns (r = 0.28, p < 0.001, Spearman correlation) than the global gradients (r = 0.06, *p* = 0.004, Spearman correlation). As for the cognition-predictive patterns, global gradients led to more consistent predictive patterns (r = 0.06, *p* = 0.003, Spearman correlation) than the local gradients (r = 0.03, *p* > 0.05, Spearman correlation). Furthermore, we examined the contribution of thalamic nuclei as defined by AAL3 atlas. For the global gradients, the anterior nucleus was the primary predictor for nearly all cognitive metrics, except for processing speed, for which the intralaminar nucleus showed the greatest contribution.

**Fig. 9.**
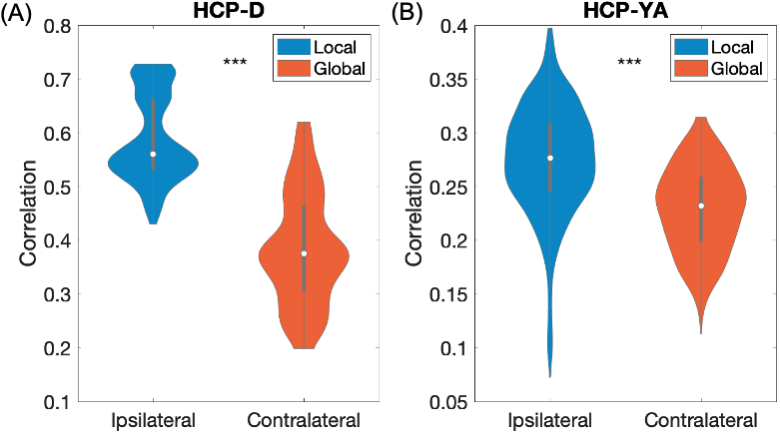
Thalamic volume prediction using ipsilateral or contralateral gradients from (A) HCP-D and (B) HCP-YA dataset. Y-axis: Spearman correlation between the true and predicted thalamic volumes. ***p < 0.001.

To ensure the specificity of the findings, we compared the gradients’ predicting thalamic volumes from the same/ipsilateral and opposite/contralateral hemisphere. We found the local gradients significantly better predicted thalamic volumes from the same hemisphere than thalamic volumes on the opposite hemisphere than the global counterparts, as shown in Figure 9.

In addition, we compared the thalamic gradients against a previously derived thalamic core-matrix gradient (Müller et al., 2020). We found that the primary anterior-posterior gradient best tracked the thalamic core-matrix gradient for both local and global gradients (HCP-D: r_local_ = 0.736, r_global_ = 0.603; HCP-YA: r_local_ = 0.626, r_global_ = 0.473).

## 4 Discussion

The thalamus, serving as a crucial hub connecting the cortex and the rest of the subcortex, plays a pivotal role in a number of cognitive processes (Aggleton & O’Mara, 2022; Hwang et al., 2022; Steiner et al., 2020) and is implicated in disorders such as schizophrenia (Pergola et al., 2015) and epilepsy (Warsi et al., 2022). Traditional analyses of thalamic functional organization often rely on FC, which typically treats the entire thalamus as a homogeneous seed while ignoring its internal spatial and anatomic variations. Emerging techniques, such as connectopic gradient mapping (Haak et al., 2018), offer a complementary representation of thalamic topography. Rather than discrete spatial boundaries, connectopic gradient mapping supposedly delineates the continuous functional topographic organization, presumably reflecting the changing connectome profiles between the seed (e.g., thalamus) and target region (e.g., neocortex). This new technique has advanced our understanding of thalamic functions in healthy (Park et al., 2024; Yang et al., 2020) and patient populations (Fan et al., 2023; Feng et al., 2025). Despite recent progress, the organization of thalamic connectome profiles across different spatial scales remains to be systematically examined. Specifically, the relationship between the local organization within the thalamus and its global thalamocortical pathways—what we term the local-global connectome profile—is not well understood. In this study, we employed the original and modified versions of the connectopic gradient mapping, and explored the local-global connectome profiles of the thalamus to discern their respective neurobiological significance. Using a predictive framework, we demonstrated that local connectome profiles encoded more information about local anatomical structure while global connectome profiles contained more information about cognition.

### 4.1 The local gradients reflect internal connectome organization while global gradients track global thalamocortical pathways

We observed a gradual change in thalamic gradients without any clear boundaries across thalamic nuclei. This observation aligns with prior work based on functional connectivity (Feng et al., 2025; Park et al., 2024; Yang et al., 2020), structural connectivity (John et al., 2025), and gene expression (Li et al., 2020; Phillips et al., 2019). By comparing the first three local (from the original connectopic mapping) and global gradients (from our rescaled approach), we showed that the two capture distinct organizational principles. The local gradients reflect the connectome organization *within* the thalamus itself, whereas the global gradients capture the connectome organization *between* the thalamus and the neocortex. Our finding remained robust regardless of the choice of the specific seed or target region chosen (for details see Supplementary Materials).

Supported by prior findings regarding primary thalamic gradients (Feng et al., 2025; Park et al., 2024; but see Yang et al., 2020), both implementations produced a similar primary gradient along the anterior-to-posterior axis (Table 1). This suggests that the principal organization of the thalamic connectome is consistent across local and global scales. This anterior-to-posterior organization has been associated with a systematic shift in function from primary functions of sensory/motor on the posterior end to higher-order cognitive functions of reward/motivation on the anterior extreme (Yang et al., 2020). Furthermore, this anterior-to-posterior gradient was linked to cytoarchitecture of the thalamus as indicated by the differential distribution of core and matrix neurons, each possessing distinct projection patterns and functional contributions (Harris et al., 2019; Jones, 2001). As for the individual-level gradients, the global gradients were much more heterogenous across participants than previously understood (Yang et al., 2020), highlighting greater inter-subject variability in the thalamus’s global connectome organization compared to its local counterpart.

### 4.2 Local and global gradients capture different aspects of thalamic characteristics

After delineating the local-global thalamic gradients, we examined their respective neurobiological relevance. Given the nature of two sets of gradients, it was expected that the local gradients would more effectively capture the local thalamic properties, such as intrinsic geometry and volume, while global gradients would better reflect individual differences in cognition. Indeed, our prediction results confirmed these expectations. This suggests that local and global thalamic gradients encode distinct types of neurobiological information, with local gradients mapping onto anatomy and global gradients linking to cognitive function.

It has been long recognized that the functional gradients are heavily influenced by the regional (Yang et al., 2020) and cortical geometry (Margulies et al., 2016). Indeed, our findings corroborated this as we showed that the intrinsic thalamic geometry exerted a greater influence on local gradients, corresponding more closely to a structural parcellation of the thalamus (AAL3 atlas). This is expected given that the local gradients reflect the local connectome profiles within the thalamus itself. For the global gradients, these anatomical constraints were relaxed but still evident. This divergence in structural constraints on functional organization across different scales aligns with a previous study that demonstrated much stronger structure-function coupling at the regional level as compared to the global, whole-brain level (Zamani Esfahlani et al., 2022). As the spatial arrangement of the cortex (and any brain region) is not arbitrary (Pang et al., 2023), our findings again underscore the critical role that thalamus’s intrinsic geometry plays in the macroscale integration and segregation of the thalamocortical pathways.

Our results also provide a deeper understanding of thalamocortical connectome profiles, highlighting their involvement in human cognition (Aggleton & O’Mara, 2022; Hwang et al., 2022; Steiner et al., 2020). For example, Steiner and colleagues examined the FC between the thalamus and major resting-state networks underlying various cognitive processes. Similar to our findings, they showed that thalamic nuclei were differentially connected to various resting-state networks, supporting diverse cognitive abilities, including processing speed, selective attention, and cognitive ﬂexibility (Steiner et al., 2020). More specifically, we found that anterior thalamus was the strongest predictor for four of the five cognitive measures: episodic memory, cognitive flexibility, inhibition control, selective attention, and total cognition. The anterior thalamus is a vital node within the Papez circuit—a loop of extended circuit involving hippocampus, fornix, mammillary bodies, cingulate gyrus, and parahippocampus—which supports episodic memory and emotion (Aggleton & O’Mara, 2022). Moreover, the anterior thalamus is implicated in attention and cognitive flexibility (Wright et al., 2015). Damage to this region can result in deficits in attentional processing (Nelson, 2021), and its dysfunction has been proposed to underlie cognitive impairments in various neuropsychiatric disorders (Roy et al., 2021).

### 4.3 Limitations and future directions

Our study is subject to several limitations that suggest avenues for future research.

First, our work focused on the thalamus to understand its local-global connectome profiles and the association with thalamus’s local characteristics and cognition. Future research should further validate whether our findings can generalize to additional structural (e.g., myelination, morphology) and cognitive metrics (e.g., emotion and language processing) as well as examine how the local-global connectome profiles of the thalamus are altered by disorders such as epilepsy, movement disorders, and schizophrenia.

Second, while we purposefully chose two preprocessing pipelines to demonstrate that our findings are robust to different methodological choices, this and cross-sectional nature of the datasets limited our ability to study the age-dependent differences in thalamic local-global connectome profiles and the thalamus’s functional specialization during development, which is subject to future investigation. Moreover, we observed overall higher consistency between the global and local gradients in the HCP-D dataset that used a larger smoothing kernel size. A large smoothing kernel size might inflate the gradient smoothness and lower the gradient’s spatial specificity. However, a small smoothing kernel, while boosting the spatial specificity, risks insufficiently denoising data, leading to reduced signal-to-noise ratio. Future studies should comprehensively evaluate the influence of different smoothing kernels on the local and global gradients.

Third, we showed the weakened anatomical constraints on thalamic gradients at the global than at the local scale. Future studies could explore the structure-function (de)coupling in other brain regions and networks (Fotiadis et al., 2024).

Lastly, in this paper we focused on the cognitive relevance of thalamic gradients and highlighted the key role the anterior thalamus plays in supporting cognition. Future work should examine the whole-brain preferential coupling patterns associated with thalamic gradients (Xie et al., 2024).

## 5 Conclusions

In this study, we explored the local-global connectome profiles of the thalamus using the original and modified connectopic mapping technique and examined the neurobiological significance of resulting functional gradients. Using two large-scale datasets, we found that its local connectome profiles primarily reflected the local anatomical structure of the thalamus, while the global thalamocortical connectome profiles were predominantly related to global cognitive processes.

## Supplementary Material

Supplementary Material

## Data Availability

The data supporting the results in this study are available within this paper and its Supplementary Materials. The HCP-D dataset is publicly available via the National Institute of Mental Health Data Archive at https://nda.nih.gov/ccf. The HCP-YA dataset is obtained from https://www.humanconnectome.org/study/hcp-young-adult. The original implementation of connectopic mapping is available at https://github.com/koenhaak/congrads, and the modified versions will be released on GitHub within 3 months following manuscript acceptance.
