# Supplementary material for: Local-global functional gradients of the thalamus capturing different aspects of thalamic structure and function
Source: Imaging Neurosci (Camb). 2026 Jan 12;4:IMAG.a.1094. doi: 10.1162/IMAG.a.1094 (PMC12797144; doi:10.1162/IMAG.a.1094)
Supplement: Supplementary Material [file IMAG.a.1094_supp.pdf]

Supplemental Materials

Mathematical derivations of different connectopic mapping implementations

As both the timeseries of seed ROI ($A \in \mathbb{R}^{t \times m}$) and the target area ($B \in \mathbb{R}^{t \times n}$) have zero mean and unit variance and we apply SVD to B so that $B = U\Sigma V^T$. We can then write the correlation matrix $C \in \mathbb{R}^{m \times n}$ as

$$C = corr(A, \tilde{B}) = A^T \tilde{B} \Sigma^{-1} = A^T U,$$

Applying cosine similarity on C, we can write the similarity matrix $S_{orig} \in \mathbb{R}^{m \times m}$ as

$$S_{orig} = cosine(C, C) = \mathrm{D}^{-1/2} C C^T \mathrm{D}^{-1/2} = \mathrm{D}^{-1/2} A^T U U^T A \mathrm{D}^{-1/2},$$

where D is a square diagonal matrix with diagonal elements of CC^Ton the diagonal.

Given the definition of SVD, U and V are unitary matrices such that $UU^T = U^T U = V^T V = VV^T = I$, where I is the identity matrix, thus we can simplify S_{orig} as

$$S_{orig} = \mathrm{D}^{-1/2} A^T A \mathrm{D}^{-1/2},$$

which is equivalent to the similarity matrix of the within-seed functional connectivity, or $corr(A, A)$.

For the gradients from the rescaled implementation, since the variance associated with singular vectors is preserved, we have

$$S_{\text{rescaled}} = \mathrm{D}^{-1/2} A^T U \Sigma^2 U^T A \mathrm{D}^{-1/2}.$$

While for the voxelwise gradients without SVD, we can rewrite its similarity matrix S_{voxel} as follows

$$S_{voxelwise} = cosine(C, C) = \mathrm{D}^{-\frac{1}{2}} A^T B B^T \mathrm{D}^{-\frac{1}{2}}.$$

Given $B = U\Sigma V^T$ and $V^T V = I$, we have

$$S_{voxelwise} = \mathrm{D}^{-\frac{1}{2}} A^T U \Sigma V^T V \Sigma \mathrm{U}^T A \mathrm{D}^{-\frac{1}{2}} = \mathrm{D}^{-\frac{1}{2}} A^T U \Sigma^2 \mathrm{U}^T A \mathrm{D}^{-\frac{1}{2}},$$

making $S_{\text{voxelwise}}$ equivalent to S_{rescaled}.

Comparing connectopic mapping implementations by randomly selecting target/seed region

To empirically validate that our results are independent from the choice of seed and target area, we also ran simulations to compare gradients from four connectopic mapping implementations. We randomly chose 1,000 voxels/vertices as the seed and

used the rest of the brain as target. We then compared similarity matrix S from different connectopic mapping implementations, since the gradients are determined by S. As shown in **Supplemental Fig. 1**, we can observe that regardless of choice of seed and target area, the similarity matrix using the original implementation closely matched that of the within-seed version, whereas the similarity matrix of rescaled implementation tracked that of the voxelwise variant.

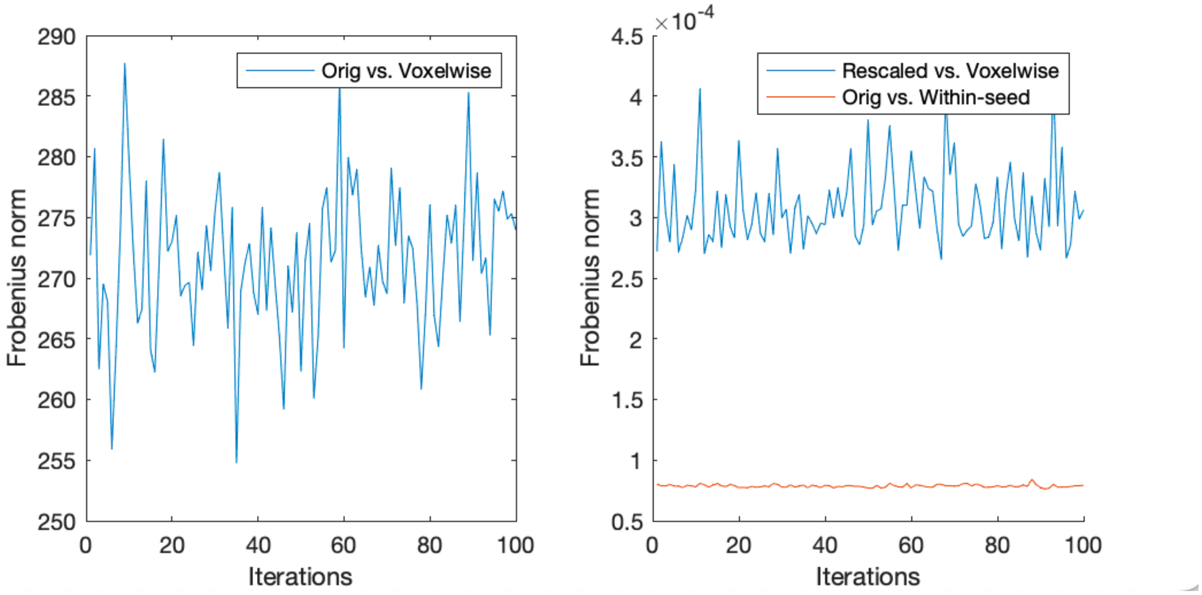

Supplemental Figure 1. Comparing similarity matrices S across connectopic mapping implementations using Frobenius norm of the difference matrix ΔS (e.g., $\Delta S = S_{voxelwise} - S_{original}$).

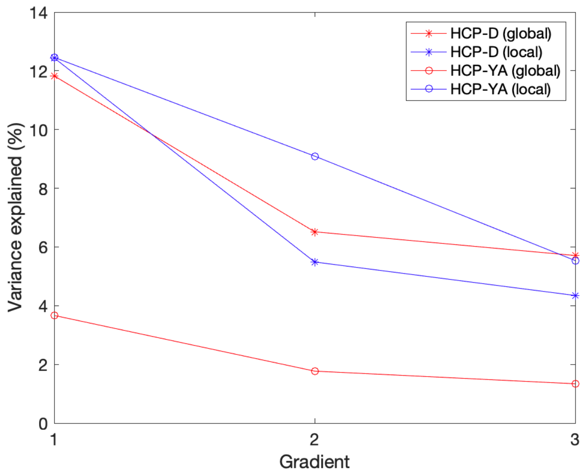

Supplemental Figure 2. Variance explained of local/global gradients using diffusion map embedding mapping.

Supplemental Table 1. Demographic information.

HCP-D	
Age (Mean ± SD, years)	14.7 ± 3.9
Sex (females/males)	324/274
HCP-YA	
Age (Mean ± SD, years)	28.8 ± 3.9
Sex (females/males)	340/258

Supplemental Table 2. Similarity of unaligned individual-level gradients across the four connectopic mapping implementations for the first three gradients DE: diffusion map embedding, LE: Laplacian eigenmap, PCA: principal component analysis.

G1 similarity (mean ± standard deviation)					
Manifold	Implementation	Original	Within-seed	Rescaled	Voxelwise
DE	Original	1±0	1±0	0.706±0.198	0.706±0.194
	Within-seed	1±0	1±0	0.706±0.198	0.706±0.194
	Rescaled	0.706±0.198	0.706±0.198	1±0	1±0
	Voxelwise	0.706±0.194	0.706±0.194	1±0	1±0
LE	Original	1±0	1±0	0.679±0.204	0.681±0.199
	Within-seed	1±0	1±0	0.679±0.204	0.681±0.199
	Rescaled	0.679±0.204	0.679±0.204	1±0	1±0
	Voxelwise	0.681±0.199	0.681±0.199	1±0	1±0
PCA	Original	1±0	1±0	0.527±0.206	0.527±0.207
	Within-seed	1±0	1±0	0.527±0.206	0.527±0.207
	Rescaled	0.527±0.206	0.527±0.206	1±0	1±0
	Voxelwise	0.527±0.207	0.527±0.207	1±0	1±0
G2 similarity (mean ± standard deviation)					
Manifold	Implementation	Original	Within-seed	Rescaled	Voxelwise
DE	Original	1±0	1±0	0.394±0.206	0.392±0.204
	Within-seed	1±0	1±0	0.394±0.206	0.392±0.204
	rescaled	0.394±0.206	0.394±0.206	1±0	1±0
	Voxelwise	0.392±0.204	0.392±0.204	1±0	1±0
LE	Original	1±0	1±0	0.387±0.202	0.382±0.197
	Within-seed	1±0	1±0	0.387±0.202	0.382±0.197
	Rescaled	0.387±0.202	0.387±0.202	1±0	1±0
	Voxelwise	0.382±0.197	0.382±0.197	1±0	1±0
PCA	Original	1±0	1±0	0.527±0.206	0.527±0.207
	Within-seed	1±0	1±0	0.527±0.206	0.527±0.207
	Rescaled	0.527±0.206	0.527±0.206	1±0	1±0
	Voxelwise	0.527±0.207	0.527±0.207	1±0	1±0
G3 similarity (mean ± standard deviation)					

Manifold	Implementation	Original	Within-seed	rescaled	voxelwise
DE	Original	1±0	1±0	0.351±0.17	0.349±0.174
	Within-seed	1±0	1±0	0.351±0.17	0.349±0.174
	rescaled	0.351±0.17	0.351±0.17	1±0	1±0
	voxelwise	0.349±0.174	0.349±0.174	1±0	1±0
LE	Original	1±0	1±0	0.343±0.167	0.336±0.165
	Within-seed	1±0	1±0	0.343±0.167	0.336±0.165
	rescaled	0.343±0.167	0.343±0.167	1±0	1±0
	voxelwise	0.336±0.165	0.336±0.165	1±0	1±0
PCA	Original	1±0	1±0	0.343±0.165	0.343±0.168
	Within-seed	1±0	1±0	0.343±0.165	0.343±0.168
	rescaled	0.343±0.165	0.343±0.165	1±0	1±0
	voxelwise	0.343±0.168	0.343±0.168	1±0	1±0
